# The Combination of the M2 Muscarinic Receptor Agonist and Chemotherapy Affects Drug Resistance in Neuroblastoma Cells

**DOI:** 10.3390/ijms21228433

**Published:** 2020-11-10

**Authors:** Anna Maria Lucianò, Elisa Perciballi, Mario Fiore, Donatella Del Bufalo, Ada Maria Tata

**Affiliations:** 1 Department of Biology and Biotechnologies Charles Darwin, Sapienza University of Rome, 00185 Rome, Italy; anna.luciano94@gmail.com (A.M.L.); perciballi.1630138@studenti.uniroma1.it (E.P.); 2 Institute of Molecular Biology and Pathology, CNR, 00185 Rome, Italy; mario.fiore@uniroma1.it; 3 Preclinical Models and New Therapeutic Agents Unit, IRCCS Regina Elena National Cancer Institute, 00144 Rome, Italy; donatella.delbufalo@ifo.gov.it; 4 Research Centre of Neurobiology Daniel Bovet, 00185 Rome, Italy

**Keywords:** neuroblastoma, M2 muscarinic receptors, drug resistance, efflux pumps, doxorubicin, cisplatin

## Abstract

One of the major limits of chemotherapy is depending on the ability of the cancer cells to elude and adapt to different drugs. Recently, we demonstrated how the activation of the M2 muscarinic receptor could impair neuroblastoma cell proliferation. In the present paper, we investigate the possible effects mediated by the preferential M2 receptor agonist arecaidine propargyl ester (APE) on drug resistance in two neuroblastoma cell lines, SK-N-BE and SK-N-BE(2C), a sub-clone presenting drug resistance. In both cell lines, we compare the expression of the M2 receptor and the effects mediated by the M2 agonist APE on cell cycle, demonstrating a decreased percentage of cells in S phase and an accumulation of SK-N-BE cells in G1 phase, while the APE treatment of SK-N-BE(2C) cells induced a block in G2/M phase. The withdrawal of the M2 agonist from the medium shows that only the SK-N-BE(2C) cells are able to rescue cell proliferation. Further, we demonstrate that the co-treatment of low doses of APE with doxorubicin or cisplatin significantly counteracts cell proliferation when compared with the single treatment. Analysis of the expression of ATP-binding cassette (ABC) efflux pumps demonstrates the ability of the M2 agonist to downregulate their expression and that this negative modulation may be dependent on N-MYC decreased expression induced by the M2 agonist. Our data demonstrate that the combined effect of low doses of conventional drugs and the M2 agonist may represent a new promising therapeutic approach in neuroblastoma treatment, in light of its significant impact on drug resistance and the possible reduction in the side effects caused by high doses of chemotherapy drugs.

## 1. Introduction

Neuroblastoma (NB) is the most common type of solid extracranial tumor, with occurrence in childhood and showing a differentiated clinical behavior [1]. The features of this neoplasia include high frequency of metastatic diffusion, early age of onset and the ability of cancer cells to resist to multiple drug therapies, a feature also known as multi-drug resistance (MDR) [2].

Although chemotherapy represents one of the most common treatments for neuroblastoma, chemoresistance tends to emerge as a major obstacle in its pharmacological treatments, as it does in many other forms of cancers [3]. Thus, despite significant advances in cancer treatment, MDR remains a major clinical issue, limiting the applicability of therapeutic protocols in several cancer types, including neuroblastoma [4].

Nowadays, we know that chemoresistance is caused by the acquisition of a phenotype commonly known as MDR, determined by multiple factors, such as quantitative or qualitative alterations of the intracellular targets alteration, in response to cytotoxic damage or activation of detoxification systems in the cells [5,6,7]. Notwithstanding what is mentioned above, chemoresistance is caused predominantly by the acquisition of cellular defense factors, including multi-drug efflux proteins. These proteins, which belong to the superfamily of ATP-binding cassette (ABC) transporters, are transmembrane proteins commonly implicated in different physiological roles and are overexpressed by tumor cells, causing a greater outflow of drugs [8,9,10]. Among these proteins, ATP-binding cassette C1 type (ABC-C1), also called multi-drug resistance proteins1 (MRP1), represents the main factor enabling the chemoresistance of several tumors, including neuroblastoma. Interestingly N-MYC [4,11], a transcriptional factor extensively involved in tumorigenesis processes [12], is one of the main regulators of a number of drug efflux pumps. It is known that MRP1 is a gene target of N-MYC and that higher MRP1 expression is frequently correlated to higher amplification and expression of N-MYC in neuroblastoma cells [13,14,15].

Studies conducted in our laboratory have demonstrated that muscarinic receptors play relevant roles both in physiological and pathological conditions [16,17,18,19,20,21]. In recent years, the implication of muscarinic receptors has been largely reported as novel therapeutic targets for the treatment of different forms of cancers [22]. It has been shown how acetylcholine (ACh), one of the main neurotransmitters in the nervous system, could be synthetized by different tumor cell types. The production of ACh by tumor cells and the subsequent interaction with muscarinic receptors frequently activate an autocrine/paracrine loop, modulating cell proliferation, migration and angiogenesis [23]. Several studies have largely reported the involvement of muscarinic receptors in several primary and metastatic tumors, such as colon [24,25], ovary [26], prostate [27,28,29], lung [30,31] and breast carcinoma [32,33,34,35], melanoma [36] and in glioblastoma, as demonstrated by our group [37,38,39,40,41,42].

More recently, we expanded this list including neuroblastoma, focusing our attention on the M2 muscarinic receptor subtype [43]. What stands out within our research is how selective activation of this receptor can counteract cell proliferation and survival. More specifically, via selective activation with the M2 agonist arecaidine propargyl ester (APE), we have observed an arrest of cell proliferation in a dose-dependent manner both in neuroblastoma and neuroepithelioma cell lines. We found that the effect produced by APE is associated with the selective activation of M2 receptors; in fact, by silencing the M2 sub-type, we observed an abolishment of the effects that APE mediated [43].

The results previously obtained have led us to investigate a possible involvement of M2 muscarinic receptors in chemoresistance processes. We used two neuroblastoma cell lines, the parental cell line SK-N-BE and one sub-clone resistant SK-N-BE(2C), characterized by a missense mutation of p53 in exon 5, with cysteine substitution in phenylalanine [44]. In these tumor cell lines we demonstrated that activation of an M2 receptor causes an arrest of the cell cycle progression and downregulates the expression of ABC efflux pumps. Moreover, we demonstrated that co-treatment with low doses of the selective M2 agonist and chemotherapy drugs currently used for neuroblastoma treatment (i.e., doxorubicin and cisplatin) significantly affects cell proliferation compared to treatment with chemotherapy drugs alone.

## 2. Results

### 2.1. Muscarinic Receptor Expression in Neuroblastoma Cell Lines

**Figure 1:**
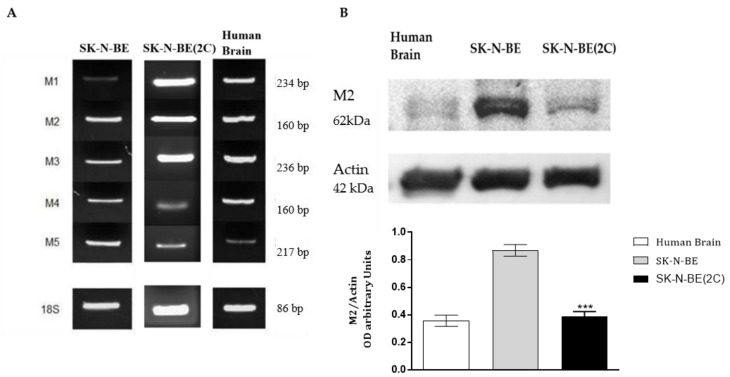
(**A**) RT-PCR analysis for all five muscarinic receptor subtypes in SK-N-BE and SK-N-BE(2C). The resistant cell line shows major expression of the odd muscarinic receptors M1 and M3. Here, 18S was used as housekeeping gene. Human brain was used as positive control. (**B**) Representative Western blot showing M2 muscarinic receptor expression in SK-N-BE and SK-N-BE(2C) cell lines. β-actin was used as protein reference. Protein extract from human brain was used as positive control. The graph below reports the densitometry of the bands for the M2 receptor protein normalized with the protein reference (actin). The data are the average of three independent experiments (*** *p* < 0.001, *t*-test; SK-N-BE(2C) vs. SK-N-BE).

We firstly evaluated the expression of muscarinic receptors in SK-N-BE and SK-N-BE(2C) cell lines by RT-PCR analysis. Both cell lines express all five muscarinic receptors at the transcriptional level, although the SK-N-BE(2C) cell line shows higher levels of the M1 and M3 subtypes (Figure 1A). Considering our interest specifically on the effects mediated by the M2 receptor, we also evaluated its expression by Western blot analysis, observing a higher expression of the M2 protein in the parental cell line (SK-N-BE) compared to the resistant line (SK-N-BE2C) (Figure 1B).

### 2.2. Analysis of Cell Proliferation

**Figure 2:**
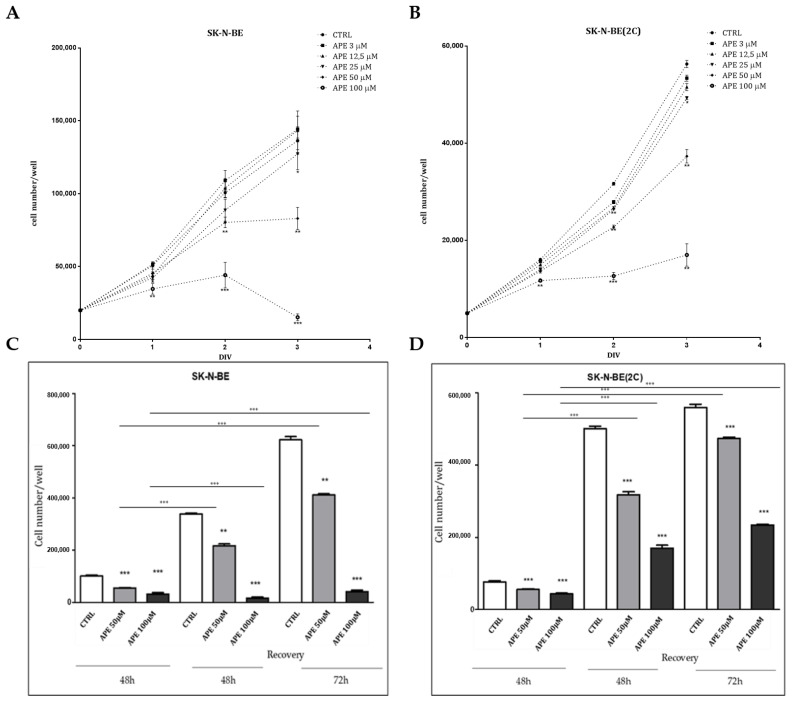
Analysis of cell growth by MTT assay in SK-N-BE (**A**) and SK-N-BE(2C) (**B**) cells upon M2 agonist APE treatment at the indicated concentrations (ranging from 3 to 100 μM) for 24, 48 and 72 h (DIV = days in vitro). The data represented are the average ± SEM of four independent experiments conducted in triplicate (* *p* < 0.05; ** *p* < 0.01; *** *p* < 0.001). An ANOVA test was used to statistically compare all experimental conditions. Recovery analysis was assessed by MTT assay in SK-N-BE (**C**) and SK-N-BE(2C) (**D**) cell lines upon 50 or 100 µM APE treatment for 48 h; then, the agonist was removed from the culture medium and the cells were maintained in a fresh complete medium for an additional 48 and 72 h. The data show the recovery of cell proliferation after 50 µM APE in both cell lines (** *p* < 0.01; *** *p* < 0.001). Instead, after 100 µM APE, only SK-N-BE(2C) was able to rescue cell proliferation (*** *p* < 0.001; APE. Respective to CTRL). The results are the mean ± SEM of at least three independent experiments conducted in triplicate. (* on the bars indicates the comparison of samples after APE withdrawal vs. respective APE treatment; *** *p* < 0.001). All the data were obtained in three independent experiments performed in triplicate.

Previous data indicated that the selective activation of M2 receptors by the agonist APE caused a decreased cell proliferation in two different neuroblastoma cell lines and in neuroepithelioma cells (43). For this reason, we compared the response of SK-N-BE and SK-N-BE(2C) cell lines, characterized by a different resistance grade to pharmacological treatments, to the M2 agonist APE, in terms of cell proliferation. By MTT assay, we demonstrated that the APE treatment was able to reduce cell growth in a dose-dependent manner (Figure 2A,B). However, the doses that most significantly inhibited cell growth were 50 and 100 µM for SK-N-BE and only the 100-µM dose for SK-N-BE(2C).

In order to demonstrate that the inhibition of cell growth was reversible, a recovery assay was performed. Cells were, thus, treated for 48 h with 50 or 100 µM APE, then the agonist was removed and a fresh medium was added to the cells. The cell number was assessed by cell count at 48 and 72 h after the M2 agonist withdrawal. Both cell lines were able to rescue the proliferation upon 50 µM APE treatment. Conversely, following treatment with 100 µM APE, cell proliferation was recovered only by SK-N-BE(2C), albeit at a lower proliferative rate than untreated cells (Figure 2C,D).

**Figure 3:**
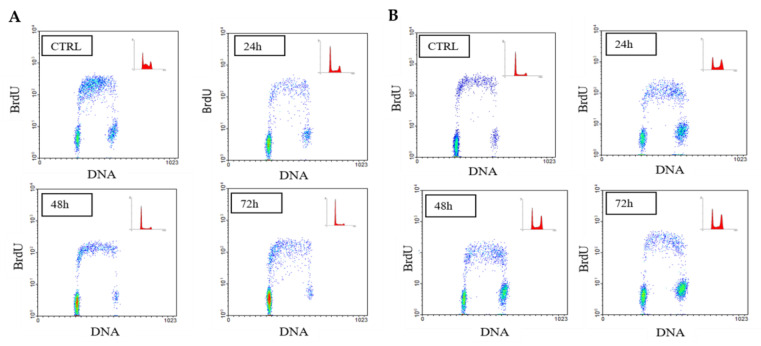
(**A**) Bivariate analysis of bromodeoxyuridine (BrdUrd) incorporation (ordinate) and DNA content (abscissa) in SK-N-BE cells and (**B**) SK-N-BE(2C) cells treated with 100 µM APE for 24–72 h. The BrdUrd-labelled cell fraction appears dramatically reduced in APE-treated cells in both cell lines.

To test our hypothesis regarding the M2 agonist’s ability to control cell proliferation in both neuroblastoma cell lines, we performed a FACS analysis in both cell lines that were kept either in the presence or absence of 100 μM APE at different experimental time points (24, 48 and 72 h). As indicated in Figure 3 and in Table 1A,B, neuroblastoma cells, following treatment with the M2 agonist APE, showed a significant reduction in the percentage of cells in S phase compared to the control condition (untreated cells). Interestingly, the SK-N-BE cells progressively accumulated in G1 phase, while the SK-N-BE(2C) did so in G2/M phase (see Table 1A,B).

**Figure 4:**
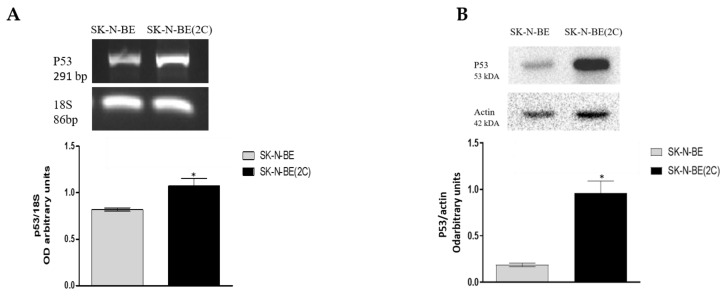
Representative RT-PCR and Western blot showing the p53 transcript (**A**) and protein (**B**) expression in SK-N-BE and SK-N-BE(2C) cells. For RT-PCR, 18S was used as a housekeeping gene; for Western blot analysis, β-actin was used as a reference protein. The graphs below report the densitometric analysis of the band of p53 normalized respectively with 18S and β-actin, obtained from three independent experiments (* *p* < 0.05; *t*-test).

To explain the cause of these different responses, especially in terms of cell cycle perturbation, we analyzed the expression of the p53 protein. As reported in Figure 4, the p53 protein resulted significantly overexpressed in SK-N-BE(2C) both as transcript (Figure 4A) and as protein (Figure 4B), while its expression is less evident in the parental cell line.

### 2.3. Chemosensitivity Test

**Figure 5:**
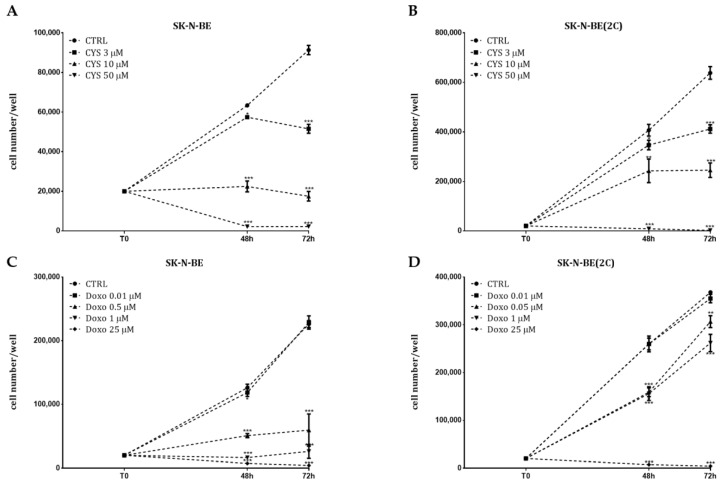
Analysis of cell proliferation by MTT assay after treatment with (**A**,**B**) cisplatin at concentrations ranging from 3 to 50 µM and (**C**,**D**) doxorubicin at concentrations ranging from 0.01 to 25 µM in SK-N-BE (**A**,**C**) and SK-N-BE(2C) (**B**,**D**). Data represented are the average ±SEM of three independent experiments conducted in triplicate (** *p* < 0.01; *** *p* < 0.001).

In order to test for potential chemoresistance, in particular for SK-N-BE(2C), we performed a chemosensitivity tests that allowed us to evaluate the pharmacological responses of both cell lines to conventional chemotherapy drugs, such as doxorubicin and cisplatin, and to further identify the minimum doses unable to produce significant changes in terms of cell proliferation. An MTT assay was performed in both cell lines in the presence of different concentrations of two drugs, demonstrating a dose-dependent effect on cell proliferation, in particular in SK-N-BE (Figure 5A,B). In fact, the SK-N-BE(2C) cells are able to respond to cisplatin treatment in a dose-dependent manner but present high resistance to doxorubicin, showing a significant reduction in cell proliferation only at 25 µM (Figure 5C,D).

However, we found that within 48 h of treatment, the doses of 3 µM cisplatin (Figure 5A,C) and 0.01 µM doxorubicin (Figure 5B,D) did not show any effects in terms of inhibition of cell proliferation in both cell lines.

**Figure 6:**
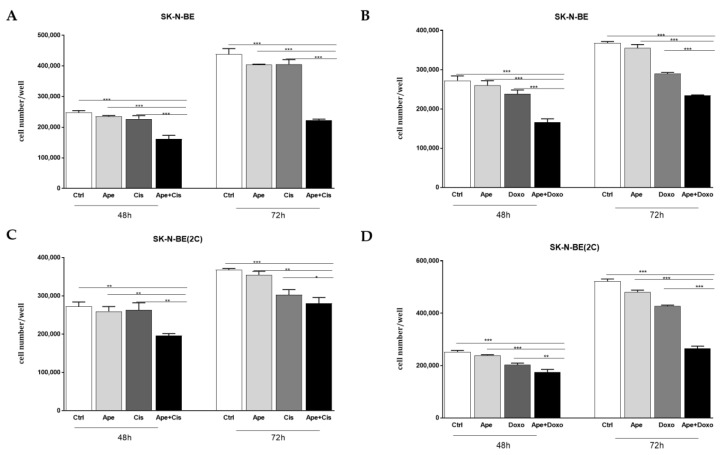
Analysis of cell proliferation by MTT assay after 48 and 72 h of co-treatment with APE and cisplatin (Cis) in SK-N-BE (**A**) and SK-N-BE(2C) (**C**) cells treated with 3 µM APE, 3 µM Cis and 3 µM APE + 3 µM Cis). (* *p* < 0.05; ** *p* < 0.01; *** *p* < 0.001; Ape + Cis vs. Ctrl; Ape + Cis vs. APE; Ape + Cis vs. Cis). Analysis of cell proliferation by MTT assay after 48 and 72 h of co-treatment with APE and doxorubicin (Doxo) in SK-N-BE (**B**) and SK-N-BE(2C) cells (**D**) treated with 3 µM APE, 0.01 µM Doxo and 3 µM APE + 0.01 µM Doxo. (** *p* <0.01; *** *p* < 0.001; Ape + Doxo vs. Ctrl; Ape + Doxo vs. APE; Ape + Doxo vs. Doxo). The data shown are the average ±SEM of four independent experiments conducted in triplicate (an ANOVA test was used to statistically compare all experimental conditions).

From the MTT assay performed in the presence of different doses of APE (Figure 2A,B), we selected a low dose of the M2 agonist APE in both cell lines (3 µM) and we tested whether this dose showed any effects when combined with low doses of both chemotherapy drugs. The MTT assay demonstrated that the single treatment of APE and chemotherapy drugs does not have any effect on cell growth within 24 h of co-treatment, but when we combined APE with one of the chemotherapy drug at low doses, we observed a significant decrease in cell proliferation compared to untreated cells and also compared to the cells treated with doxorubicin or cisplatin alone (Figure 6).

### 2.4. M2 Agonist Modulates the Efflux Pumps Expression

**Figure 7:**
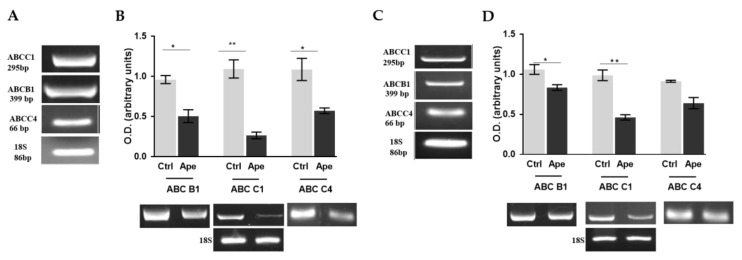
RT-PCR analysis showing the ABC-B1, ABC-C1 and ABC-C4 transcript levels in SK-N-BE (**A**) and SK-N-BE(2C) cell lines (**C**). Here, 18S used as housekeeping gene. RT-PCR analysis showing the ABC-B1, ABC-C1 and ABC-C4 transcript levels in SK-N-BE (**B**) and SK-N-BE(2C) (**D**) cell lines after 24 h of treatment with 100 µM APE. The graphs indicate the densitometric analysis of the bands of the transcript normalized with the housekeeping gene 18S. The results are the average ± SEM of three independent experiments performed in triplicate. (* *p* < 0.05; ** *p* < 0.01; *t*-test; APE vs. respective Ctrl).

Considering the ability of M2 agonists to strengthen the inhibitory effects of chemotherapy drugs on neuroblastoma cell proliferation when low doses were used, we further evaluated the ability of the M2 agonist to modulate the drug efflux pumps in both cell lines. At first, using RT-PCR analysis, we demonstrated that both neuroblastoma cells expressed the ABC-B1, ABC-C1 and ABC-C4 pumps at transcriptional levels (Figure 7A,C).

**Figure 8:**
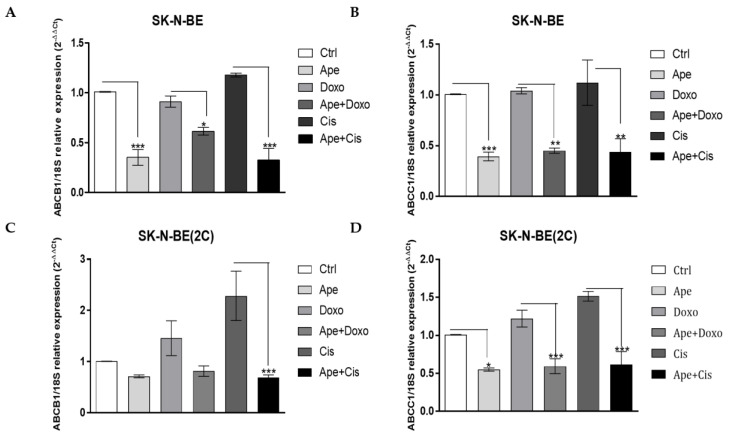
qRT-PCR analysis showing the expression of ABC-B1 in SK-N-BE (**A**) and SK-N-BE(2C) (**C**) cells treated with APE 3 µM, Doxo 0.01 µM, APE 3 µM + Doxo 0.01 µM, Cis 3 µM and APE 3 µM + Cis 3 µM. qRT-PCR analysis showing the expression of ABC-C1 in SK-N-BE (**B**) and SK-N-BE(2C) (**D**) cells treated with APE 3 µM, Doxo 0.01 µM, APE 3 µM + Doxo 0.01 µM, Cis 3 µM and APE 3 µM + Cis 3 µM. The data are the average ±SEM of four independent experiments performed in duplicate; a *t*-test was used to statistically compare the different experimental conditions (* *p*< 0.05; ** *p* < 0.01; *** *p* < 0.001).

The treatment with 100 µM APE was able to significantly downregulate the expression of all three pumps in SK-N-BE (Figure 7B), while only the ABC-B1 and ABC-C1 pumps resulted downregulated in the SK-N-BE(2C) cell line (Figure 7D). Interestingly, the same effects were observed when we moved to the expression detected by qRT-PCR analysis using the minimum concentration of APE (3 µM). In all cases, we observed that the expression of ABC-B1 (Figure 8A,C) and ABC-C1 (Figure 8B,D) was significantly downregulated by APE, both when used alone and when APE was added to the chemotherapy drugs. Any effect on efflux pump expression was observed when low doses of chemotherapy drugs were used alone (Figure 8).

**Figure 9:**
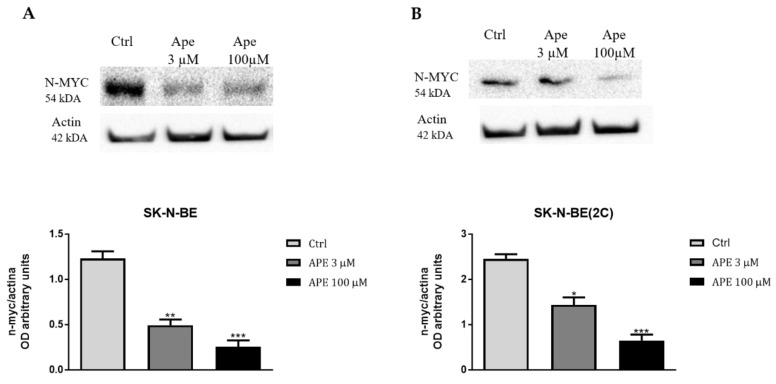
Western blot analysis of N-MYC protein expression after 3 or 100 µM of APE treatment in SK-N-BE (**A**) and SK-N-BE(2C) cells (**B**). β-actin was used to normalize the amount of total proteins loaded in each lane. The graph below reports the densitometric analysis of the band of N-MYC normalized with β-actin, obtained from three independent experiments (* *p* < 0.05; ** *p* < 0.01; *** *p* < 0.001; *t*-test APE vs. ctrl).

In order to explain how APE was able to control ABC pump expression, we analyzed the expression of one of the main oncogenes involved in neuroblastoma tumorigenesis, the proto-oncogene N-MYC, considered the main transcription factor involved in the regulation of ABC pumps [12,13,14]. By Western blot analysis, we demonstrated that both the higher and the lower doses of the M2 agonist APE were able to downregulate the expression of N-MYC in both neuroblastoma cell lines (Figure 9A,B).

## 3. Discussion

The role of M2 muscarinic receptors has been largely investigated by our group in recent years, demonstrating its ability to inhibit cell proliferation and survival in primary glioblastoma cell lines [37,38,39,40,41,42] as well as in neuroblastoma [43]. More recently, we have demonstrated that the M2 agonist APE was able to counteract drug resistance in breast cancer, demonstrating the strategic role of the M2 receptor in the therapeutic treatment of different tumor types [35]. Based on these findings, the purpose of the present work was to better investigate the role of M2 muscarinic receptor in the control of cell proliferation and chemoresistance in neuroblastoma. To this end, we decided to use two NB cell lines, the SK-N-BE and SK-N-BE(2C) cell lines, derived from the same patient after 5 months of therapy and presenting high resistance to chemotherapy drugs [44]. The two cell lines differ in p53 expression; in fact, SK-N-BE cells present the p53 wild-type, while SK-N-BE(2C) cells present a mutated p53 [44]. We have confirmed the expression of the p53 protein in both cell lines, demonstrating that the SK-N-BE(2C) showed higher levels of p53 protein than SK-N-BE, levels that may be dependent on an accumulation of the mutated form of the protein in the SK-N-BE(2C) (see Figure 4). Our results is in agreement with the data reported by Tweddle et al. demonstrating that under normal condition the parental cell line SK-N-BE shows lower expression of the P53 protein in the nucleus while the SK-N-BE(2C) have high baseline accumulation of nuclear mutant p53 [44].

The different expression of muscarinic receptors in two different cell lines by RT-PCR analysis were also analysed, demonstrating that the two cell lines express all muscarinic receptor subtypes, albeit different levels of expression are observable. The M1 and M3 receptors, muscarinic receptor subtypes generally promoting tumor cell proliferation [22], are expressed at higher levels in SK-N-B(2C) than in parental cell lines (Figure 1A). Interestingly the M2 receptors analysed at protein levels, appears significantly less abundant in SK-N-B (2C) than in SK-N-BE (Figure 1B).

Considering the differences in terms of drug resistance and of the M2 receptor expression of the two NB cell lines, we analysed the ability of the preferential M2 agonist APE to counteract cell growth. The ability of APE to selectively bind the M2 receptors was previously demonstrated by pharmacological binding experiments and silencing of the receptors both in glioblastoma [38,39]; and neuroblastoma cells [43]. The analysis by MTT assay has clearly demonstrated the only high doses of APE are able to impair cell growth in both cell lines and that SK-N-BE(2C) differently by SK-N-BE resulted unable to respond to low doses of M2 agonist (Figure 2A,B). Moreover, both cells lines are able to recover cell proliferation after that 50 µM APE was removal from the culture medium; instead when the M2 agonist was used at 100 µM concentration, only SK-N-BE(2C) was able to recover cell proliferation, differently from SK-N-BE that showed an irreversible arrest of cell growth (Figure 2 C,D). These data may contribute to support the drug resistance of SK-N-BE(2C).

In order to better understand the effects of APE on cell proliferation, we have analyzed, by FACS analysis, the cell cycle progression in the two cell lines. Interestingly, the M2 agonist treatment (100 µM) was able to arrest cell cycle progression in both cell lines causing a significant decrease in cells in S phase. However, we observed that while the SK-N-BE cells resulted preferentially accumulated in G1 phase, the SK-N-BE(2C) cells were accumulated in G2/M phase (Figure 3 and Table 1). The differential behavior of the SK-N-BE(2C) cell line may be dependent on the expression of the mutated form of p53. In fact, similar results were observed also in the glioblastoma cell line U251 and the GB8 primary glioblastoma cell line, both expressing p53 mutated [38,42]; in both cell lines, the APE treatment caused a progressive accumulation of cells in G2/M phase, suggesting that the tumor cells presenting p53 mutated present a univocal response to APE, regardless of the type of tumor.

Recent evidence indicates that metronomic poly-chemotherapy may represent a new generation of chemotherapy treatment showing more efficacy and reduced side effects. In our previous study, we demonstrated the ability of breast cancer to respond to low doses of muscarinic agonists in combination with low doses of paclitaxel [35]. Starting from this evidence, we tested the ability of low doses of the M2 agonist APE to affect neuroblastoma cell growth when in combination with conventional chemotherapy drugs. To perform this, we previously evaluated the chemosensitivity of two NB cell lines, analyzing the cell growth by MTT assay at different concentrations of cisplatin and doxorubicin. The data obtained have demonstrated that SK-N-BE cells respond to cisplatin and doxorubicin in a dose-dependent manner. Meanwhile, the SK-N-BE(2C) cells responds to cisplatin but present a greater resistance to doxorubicin (Figure 5). However, analysis of the response to these two drugs has allowed for identifying the low doses to use in combination with low doses of APE.

The metronomic combination of the drugs has clearly demonstrated that a low dose of APE with low doses of the chemotherapy drugs significantly decreased cell growth, while when low doses of the drugs were used alone, it did not show any effects on neuroblastoma cell growth (Figure 6). In order to try to explain the mechanism through which APE increases sensitivity to low doses of the chemotherapy drugs, we have analyzed the expression of multi-drug efflux pumps. In fact, one of the main mechanisms involved in chemoresistance is given by the overexpression of the multi-drug efflux pumps in tumor cells, which can increase the outflow of drug from the cells. First of all, we have characterized the main transporters involved in MDR, demonstrating that both cell lines express ABC-B1, ABC-C1 and ABC-C4 pumps at transcriptional levels (Figure 7A,C). Moreover, we also demonstrated that a high dose of APE (100 µM) was able to downregulate the expression of all pumps analyzed in SK-N-BE cells, while only the expression of the ABC-B1 and ABC-C1 pumps was reduced in SK-N-BE(2C) (Figure 7B,D). Considering that only ABC-B1 and ABC-C1 were downregulated in both cell lines, the expression of these two pumps was also evaluated after treatment with low doses of APE and chemotherapy drugs. The results obtained have clearly indicated that only APE was able to reduce the expression of the pumps when used at a low dose, while cisplatin and doxorubicin, when used at low doses, did not show any effect or even favor the increase of the expression of these two pumps in both cell lines (Figure 8). Interestingly, when APE is used in combination with the low dose of the two chemotherapy drugs, a significant reduction in expression of both ABC pumps in the two neuroblastoma cell lines is evident. This result suggests that the M2 agonist is capable of reducing the expression of drug efflux pumps, favoring the chemotherapy drugs entering and remaining, blocking the inside of the cells, thus allowing an accumulation of the drugs into the cells and increasing their toxic effects also when they are used at low doses.

Finally, in order to identify possible mechanisms that could explain the ability of APE to reduce the expression of the efflux pumps, we have analyzed the effect of APE on the expression of N-MYC, the main transcriptional factor involved in the onset and progression of neuroblastoma [12] and able to act as a transcriptional factor in the regulation of ABC pumps, especially for ABC-C1. After treatment with high and low doses of APE (100 and 3 µM), the expression of N-MYC protein was significantly reduced (Figure 9). The ability of APE to downregulate N-MYC expression may explain its effect both on the decreased expression of efflux pumps and on neuroblastoma cell proliferation inhibition.

## 4. Materials and Methods

### 4.1. Cell Cultures

Human neuroblastoma cell lines SK-N-BE and SK-N-BE(2C) were cultured in Roswell Park Memorial Institute (RPMI) 1640 medium (St. Louis, MO, USA, Sigma) supplemented with 10% fetal bovine serum (FBS) (Immunological Sciences, Rome, Italy), 50 mg/mL streptomycin, 50 IU/mL penicillin and 2 mM glutamine (Sigma) and maintained at 37 °C in atmosphere of 90% O_2_ and 5% CO_2_.

### 4.2. Cell Viability Assays

Cells were seeded on 24-well plates at a density of 2 × 10^3^ cells/well. After 24 h, cells were treated with the cholinergic agonist arecaidine propargyl ester hydrobromide (1-Methyl-1,2,5,6-tetrahydro-3-pyridine carboxylic acid propargyl ester hydrobromide; APE) (Sigma Aldrich, St. Louis, MO, USA) at different times (ranging from 24 to 72 h). Cell proliferation was assessed by colorimetric assay based on 3-(4.5-dimethyl thiazol 2-y1)-2.5-diphenyl tetrazolium bromide (MTT, Sigma-Aldrich, St. Louis, MO, USA) metabolization. The MTT assay was performed according the protocol optimized by Mosmann [45]. MTT was dissolved in PBS at 5 mg/mL. The stock of MTT solution (10X) was added and diluted (1X) in each well and then incubated at 37 °C for 3 h. Isopropanol was added to all wells and mixed thoroughly to dissolve the dark blue crystals. For each well, the OD at 570 nm was measured by the GloMax Multi Detection System (Promega, Madison, WI, USA).

### 4.3. Recovery Assay

To assess whether inhibition of cell proliferation—APE induced was reversible, a recovery analysis was set up. The cells were treated for 48 h with APE. Then, the medium containing APE was withdrawn, the cells were washed with PBS and fresh complete medium without APE was added. Proliferation analysis was assessed by MTT assay (Sigma-Aldrich, St. Louis, MO, USA). For each well, the OD at 570 nm was measured by the GloMax Multi Detection System (Promega, Madison, WI, USA) at different time points, after 48 and 72 h, after APE withdrawal.

### 4.4. Flow Cytometry Assay

The cells were plated on 60-mm diameter dishes at a density of 2 × 10^5^ cells/dish. The day after plating, the cells, excluding control samples, were treated with 100 μM APE for 24, 48 and 72 h. At the end of the treatment, cells were incubated for 60 min with 45 µM bromodeoxyuridine (final concentration) (BrdUrd, Sigma-Aldrich, St. Louis, MO, USA), collected by trypsinization, centrifuged for 10 min at 1000 rpm and then fixed in methanol/PBS 1:1 (*v*/*v*). To identify the cells in S phase, DNA content and BrdUrd incorporation were determined in simultaneous analyses by staining with propidium iodide (PI) and anti-BrdU, respectively. Partial DNA denaturation was performed by incubating the cells in 3N HCl for 45 min, followed by neutralization with 0.1 M sodium tetraborate. Samples were then incubated with monoclonal anti-BrdU (1:50 *v*/*v*; Dako, Santa Clara, CA, USA; RRID:AB_10013660) for a further 30 min at room temperature (RT), washed twice with 0.5% Tween-20 in PBS and incubated for 30 min with anti-mouse Alexa 488 fluor-conjugated (1:600) (Promega Italia, Milan, Italy). After washing samples with PBS plus 0.5% Tween-20, they were stained with 10 µg/mL PI for 15 min at room temperature (RT). Flow cytometry analysis was performed with a flow cytometer Coulter Epics XL with 488 nm wavelength excitation and 10^4^ events were collected for each sample. Biparametric (DNA content vs. BrdU content) analysis was performed using WinMDI 2.7 software.

### 4.5. Chemosensitivity Test

In order to evaluate the chemosensitivity of both cell lines to different concentrations of the chemotherapy drugs, we performed chemo-sensitivity tests, treating cells with decreasing doses of cisplatin and doxorubicin. In this regard, our experiments were based on the treatment of SK-N-BE cells and SK-N-BE(2C) cells with decreasing concentrations of doxorubicin (range 25–0.01 μM) and cisplatin (range 25–3 μM) for 24, 48 72 h. The data obtained allowed to identify the lower concentration that showed reduced effects on cell proliferation in both cell lines. Therefore, for the following experiments, we decided to select the concentration of 0.01 μM for doxorubicin and 3 µM for cisplatin within the 24 h of treatment.

### 4.6. Western Blot

Cells were lysed with lysis buffer (Tris 10 mM, NP40 0.5%, NaCl 150 mM) containing protease inhibitors (50×; Sigma-Aldrich, St. Louis, MO, USA). The total amount of protein loaded for Western blot was 30 or 40 μg (depending on samples and protein analyzed). After protein extraction, the quantification of the total amount of protein was determined by the Pierce™ BCA Protein Assay Kit (Thermo Fisher Scientific) according to the manufacturer’s protocol. Then, the sample buffer supplemented with 5% β-mercaptoethanol was added to protein lysates and heated for 5 min at 95 °C. Protein extracts were loaded on 10% SDS-polyacrylamide gel (PAGE) and transferred to PVDF membrane (Merck Millipore, KGaA, Darmstadt, Germany). Membranes were blocked for 1 h in 5% non-fat milk powder (Sigma-Aldrich, St. Louis, MO, USA) in PBS containing 0.1% Tween-20 (Sigma-Aldrich, St. Louis, MO, USA) and then incubated with monoclonal anti-M2 antibody (1:800 Abcam, Cambridge, UK AB_2251128), polyclonal anti-N-MYC antibody (1:500 Santa Cruz Biotechnology, Texas, USA; sc-791) and monoclonal anti-p53 antibody (1:100 Santa Cruz Biotechnology, Texas, USA, AB_628082) overnight at 4 °C. Blots were washed with PBS + Tween and then incubated for 1 h with anti-mouse secondary antibody horseradish-peroxidase-conjugated (Promega Italia, Milan, Italy). Signal was revealed by Chemiluminescence (ECL) reagents (Immunological Science, Milan, Italy). Immunoreactive bands were visualized in a Chemidoc system (Molecular Imager ChemiDoc XRS + System with Image Lab Software, Biorad, CA, USA) and the optical density of the bands was quantified by ImageJ software (National Institutes of Health, NIH, 469 Bethesda, MD, USA). Beta-actin was used as protein reference for loading control (monoclonal anti β-actin antibody (1:800 Sigma-Aldrich, St. Louis, MO, USA, A5441).

### 4.7. RNA Extraction RT-PCR and qRT-PCR Analysis

Total RNA was extracted using the Total RNA extraction Mini Kit (FMB, Philadelphia, PA, USA), following the manufacturer’s instructions, and then digested with DNAse I (Ambion-Life Technologies Italia, Monza, Italy). For each sample, 1 μg of total RNA was reverse transcribed for 60 min at 37 °C with Random Primers (Promega, Madison, WI, USA) and M-MLV reverse transcriptase (Promega, Madison, WI, USA). Primers and GoTaq Green Master Mix (Promega, Madison, WI, USA) were added to 100 ng of cDNA. The expression of all the five muscarinic receptors transcripts was evaluated by semi-quantitative RT-PCR analysis using the following primers:

- CHRM1: (Acc. N. AW49504)

F 5′-AGAGAGACCCTGCCAACTTT-3′

R 5′-CTCCTGACTTTCCTGCCTAAA-3′

- CHRM2: (Acc. N. NM_000739.3)

F 5′-CTCCAGCCATTCTCTTCTGG-3′

R 5′-CTCCAGCCATTCTCTTCTGG-3′

- CHRM3: (Acc. N. NM_000740.4)

F 5′-CGCTCCAACAGGAGGAAGTA-3′

R 5′-GGAGTTGAGGATGGTGCTGT-3′

- CHRM4: (Acc. N. NM_000741.5)

F 5′-AATGAAGCAAGAGCGTCAAGAA-3′

R 5′-TCATTGGAAGTGTCCTTATCA-3′

- CHRM5 (Acc. N. NM_001320917.2)

F 5′-CCTGGCTGATCTCCTTCATC-3′

R 5′-GTCCTTGGTTCGCTTCTCTG-3′

- TP53 (Acc. N. NM_000546.6)

F 5′-CCCCTGTCATCTTCTGTCCCTT-3′

R 5′-CAGACCATCGTCATCTGAGCAG-3′

- 18S (Acc. N. NR_1461152)

F 5′-CCAGTAAGTGCGGGTCATAAGC-3′

R 5′-AACGATCCAATCGGTAGTAGCG-3′

RT-PCR starts with denaturation step at 95 °C for 4 min, followed by 25 cycles of amplification at 60 °C for 30 s (18 s gene), 32 cycles at 60 °C for 30 s (M1-M5 muscarinic receptors) or 34 cycles at 64 °C for 30 s (ABC pumps) and final extension at 72 °C for 30 s.

The expression of the efflux pumps was also analyzed by qRT-PCR. For qRT-PCR, a quantity of 50 ng of each cDNA was used as a template in each tube for real time-PCR assay. SYBER green PCR Master Mix (Applied Biosystems Italia, Monza, Italy) (final concentration 1X) and primers (final concentration of 0.4 mM) were also added at the respective reaction tubes and analyzed by Thermofisher Quantstudio3. All samples were run in triplicate. The real-time PCR conditions included a denaturing step at 95 °C for 3 min followed by 40 cycles at 95 °C for 30 s, 60 °C for 30 s and 75 °C for 45 s. Two cycles were included as final steps: one at 95 °C (1 min) and the other at the annealing temperature specific for each couple of primers used (1 min).

Quantification was expressed as 2^−ΔΔCT^ where ΔΔCT = ΔCTsample − ΔCTcalibrator.

The primers used for qRT-PCR are the following:

- ABCB1: (Acc. N. NM_000927.5)

F 5′-CGACAGGAGATAGGCTGGTT-3′

R 5′-AGAACAGGACTGATGGCCAA-3′

- ABCC1: (Acc. N. XM_017023237.1)

F 5′-TGCTCACTTTCTGGCTGGTA-3′

R 5′-ACAGGACAAGACGAGCTGAA -3′

- ABCC4: (Acc. N. NM_001105515.3)

F 5′- AGACCCCAACTCTACAAGGC-3′

R 5′-ATTCTTCCATGCACGCTGAC-3′

### 4.8. Statistical Analysis

The Student’s *t*-test and one-way ANOVA test followed by Bonferroni’s post-test were used to evaluate statistical significance within the different samples. Results were considered statistically significant at *p* < 0.05 (*), *p* < 0.01 (**) and *p* < 0.001 (***).

## 5. Conclusions

The data reported in the present work, together with the results obtained in other tumor types [37,38,39,40,41,42], clearly confirm the M2 receptor as a new and interesting therapeutic tool to counteract tumor cell proliferation and survival. Moreover, the results obtained in neuroblastoma and in breast cancer [35,43] suggest a strategic role of the M2 receptor in counteracting drug resistance in different tumor types. In fact, the combined effect of low doses of the M2 agonist and chemotherapy drugs results in potentiating the toxic effect of the drugs to low doses through the decreased expression of MDR pumps. These results suggest that the drug-combined treatment may represent a new, promising therapeutic protocol for cancer treatment, contributing to increase the efficacy of chemotherapy and reducing side effects in patients.

## Figures and Tables

**Table 1:** (**A**) Percentage of SK-N-BE cells in G1, S and G2/M phases after 100 µM APE treatment. The data are the mean ±SEM. *p* values are also reported (APE-treated cells vs. control). (**B**) Percentage of SK-N-BE(2C) cells in G1, S, G2/M phases after 100 µM APE treatment. The data are the mean ±SEM. *p* values are also reported (APE-treated cells vs. respective control).

(**A**)									
**SK-N-BE**	**%G1**	**±SEM**	***p***	**%S**	**±SEM**	***p***	**%G2/M**	**±SEM**	***p***
CTRL	33.2	0.9815		52.25	2.569		12.55	3.551	
APE 24 h	63.2	1.039	0.0001	28.85	0.7217	0.0007	7.95	0.3175	0.2665
CTRL 48 h	45.55	2.223		50.3	2.309		4.15	0.8666	
APE 48 h	78.85	1.992	0.0005	17	17.312	0.0003	7.15	0.2598	0.0004
CTRL 72 h	58.6	0.4619		32.55	1.415		8.85	0.9526	
APE 72 h	82.6	0.4819	0.001	14.45	0.5485	0.0003	2.95	0.866	0.0035
(**B**)									
**SK-N-BE(2C)**	**%G1**	**±SEM**	***p***	**%S**	**±SEM**	***p***	**%G2/M**	**±SEM**	***p***
CTRL	40.4	0.6		49.9	1.155		9.7	0.8083	
APE 24 h	47	0.6	0.0166	41.3	0.0333	0.0628	11.7	0.3464	0.0853
CTRL 48 h	32.8	2.2		58.75	3.118		8.45	0.8083	
APE 48 h	40	2.4	0.0644	30.55	1.819	0.0025	29	0.7217	0.0058
CTRL 72 h	35.45	0.95		58.85	0.8372		5.7	0.1155	
APE 72 h	48	2.13	0.0025	22.75	2.281	0.0006	29.25	0.1443	0.0004
